# Assessment of Plumage and Integument Condition in Dual-Purpose Breeds and Conventional Layers

**DOI:** 10.3390/ani7120097

**Published:** 2017-12-12

**Authors:** Mona Franziska Giersberg, Birgit Spindler, Nicole Kemper

**Affiliations:** Institute for Animal Hygiene, Animal Welfare and Farm Animal Behaviour, University of Veterinary Medicine Hannover, 30173 Hannover, Germany; birgit.spindler@tiho-hannover.de (B.S.); nicole.kemper@tiho-hannover.de (N.K.)

**Keywords:** animal welfare, on-farm assessment, laying hens, feather pecking, cannibalism

## Abstract

**Simple Summary:**

Behavioural problems, such as injurious pecking, are major welfare concerns in laying hen husbandry. To take adequate measures at the right time, useful information about the occurrence of these problems in a flock can be obtained by examining the hens for feather loss and skin injuries. Although feather loss and injuries can also result from mechanical abrasion or health issues, they provide strong evidence of behavioural problems in a flock, particularly when observed on the back and the tail of the animals. In our study, the behaviour of two genetic strains of laying hens (conventional layers and dual-purpose breeds) was evaluated by means of two different methods for assessing the feathers and the skin on distinct body parts of the animals. One method was a mere visual inspection of the flocks, whereas the other included the capture and handling of individual hens. Damaging behaviour, which resulted in severe feather loss and skin injuries, only occurred in the conventional layers. Both of the methods provided similar results for feather loss and injuries for most of the tested body regions and weeks. Therefore, the mere visual method was sufficient to detect injurious behaviour in laying hens.

**Abstract:**

The assessment of plumage and integument condition in laying hens provides useful information about the occurrence of feather pecking and cannibalism. Although feather loss and skin injuries can result from mechanical abrasion or clinical diseases, they are valid animal-based indicators for behavioural disorders. This particularly applies to damage on the back and tail region of the hens. The aim was to evaluate the behaviour of dual-purpose breeds (Lohmann Dual, LD) and conventional layer hybrids (Lohmann Brown plus, LB+), and to compare a mere visual assessment (Visual S**c**oring, VSc), with a method involving the handling of individual animals (Hands-on S**c**oring, HSc). During weekly VSc, the hens’ plumage and integument were scored on five body parts. HSc was carried out on seven study days applying the same scoring scale as for VSc. In LB+ hens, minor plumage damage started at 25 weeks and increased to the 71st week. With 99.5% of LB+ showing feather loss to a different extent, the back was the most severely affected body part. In contrast, only between 4.5% and 7% of LD showed minor feather loss at the end of the study. Integument damage reached a peak, with 6% affected LB+ in week 66. Injuries were found only sporadically in LD hens. Spearman’s rho for the comparison of plumages scores given in VSc and HSc was >0.90 (*p* < 0.01) in both hybrids for most of the tested body regions and weeks, except for the breast/belly region. However, VSc and HSc were equally valid for detecting skin injuries of all of the body regions (*r*_s_ > 0.86, *p* < 0.01). Damaging behaviour only occurred in the LB+ flocks, though both of the genetic strains were kept under the same conditions. The visual scoring method was suitable for detecting both plumage and integument damage.

## 1. Introduction

Behavioural disorders, such as feather pecking and cannibalism, which cause severe damage to conspecifics and high mortality [1], are a major challenge and a serious welfare issue in modern laying hen husbandry. These problems (re-)emerged particularly after the ban on conventional cages in the European Union [2,3], and the (future) abolition of beak trimming in many European countries [4].

The term “feather pecking” refers to the non-aggressive pecking at or plucking and eating of the feathers of conspecifics [5]. Feather pecking, which is mainly directed at the birds’ back, tail, and vent area [3], is related to abnormal feeding and foraging behaviour [3,6,7]. It seems to occur particularly in situations in which the animals fail to cope with environmental stressors [3]. This behavioural disorder is clearly distinguishable from aggressive pecking, which is directed at the head/neck region, and is used to establish and maintain dominance hierarchies in groups of birds [3,5]. Some authors [5,8] defined different forms of feather pecking (i.e., gentle and severe feather pecking) according to the force of the pecks or the complete removal of feathers. However, the distinction between these types of feather pecking and tissue pecking is blurred [9]. Gentle feather pecking can turn into severe feather pecking, which can develop into tissue pecking and cannibalism as soon as denuded areas occur on the hens’ bodies [5]. In contrast, cannibalistic behaviour that is directed at the vent or the toe of a conspecific often occurs independently of feather pecking in fully feathered flocks [5,10,11].

Abnormal behaviour in laying hens can be observed in all kinds of housing systems [1,12,13], including organic farms [14]. However, it may be more difficult to control for these abnormalities in loose housing systems where feather pecking and cannibalism can be spread by social learning [15] or by damaged feathers becoming an attractive target for sustained pecking behaviour [16]. In addition, many potential victims are exposed to one offending bird in non-cage systems [9]. The causations of feather pecking and cannibalism are multifactorial, and a wide range of influencing factors have been identified, both under experimental [17,18] as well as observational conditions [13,14,19]. These factors include several management and housing aspects, for instance the availability of litter and other foraging materials in the rearing- and the laying period, the provision of an adequate diet, and light quality and intensity [4]. Furthermore, some layer strains are more likely to develop feather pecking and cannibalism than others [20,21], indicating a genetic background for these behavioural traits [9]. Investigations have been performed concerning the phenotypic selection on feather pecking in social contexts [22]. In addition, methods of molecular genetics have been studied, such as identifying quantitative trait loci for feather pecking behaviour [23]. However, these studies were carried out either in experimental layer lines [22,23] or in commercially available high yielding hybrids [24]. Little is known about the incidence of feather pecking and cannibalism in purebred laying hens [24,25] or alternative hybrids like dual-purpose hens.

It is important to notice that even under optimal conditions, the risk of the occurrence of feather pecking and cannibalism cannot be eliminated completely in practice [4]. Competent, regular monitoring and surveillance of the flocks are crucial to recognize emerging problems at once and to intervene in due time [4]. However, direct behavioural observations and video analyses are often time-consuming and difficult to perform at flock level. In contrast, the assessment of plumage and integument condition as animal-based indicators provides a feasible option to gain information about the occurrence of feather pecking and cannibalism in laying hens [26]. Plumage and integument damage can also result from mechanical abrasion or clinical diseases, such as diarrhea or nutrient deficiency. Feather damage on the underside of the hens’ neck, for instance, may be attributed to abrasion from the feeders [26]. However, feather pecking behaviour is strongly related to feather loss and skin injuries, particularly on the rump and back [26]. Although a large number of varying assessment methods have been published over the past years (e.g., [1,13,26]), it is possible to differentiate between mere visual scoring methods (VSc) and close inspections requiring capture and handling of individual birds (HSc). Since HSc methods involve catching, they may be potentially stressful for the animals and time-consuming when performed regularly on-farm, whereas VSc methods bear the risk of missing slight plumage and integument damage [27]. Therefore, a VSc method using a five point scale for five body regions was validated by means of correlating the VSc scores with scores being recorded by an HSc method [27]. In addition, inter-observer reliability was tested for the VSc method. The authors concluded that the VSc method was suitable for detecting feather damage in adequate detail, and that inter-observer reliability was high [27]. However, the same individual hens were scored by the VSc and the HSc method, and by the different observers. Additionally, it would be important to know if both of the methods also provided equally valid results at flock level, where the respective sample size might comprise different individuals on each assessment day. Though useful recommendations were made concerning the number of hens that should be examined, no information was given on how to choose these animals from the flock representatively. Furthermore, integument damage was not assessed [27].

The aim of the present study was to develop and validate a mere visual scoring method (VSc) for obtaining animal-based indicators concerning feather pecking and cannibalism in laying hens. Since this method should be feasible for regular flock monitoring and longitudinal on-farm research, it was applied to conventional layer hybrids and dual purpose breeds on a commercial scale. To test the reliability of the VSc method in this context, it was compared to a scientifically established HSc method at the flock level. It was hypothesized that in both hybrids low plumage and integument scores (i.e., no feather loss/no integument damage) in VSc would be accompanied by low scores in HSc, and that the same would be true for high scores (i.e., severe feather loss/severe integument damage).

## 2. Materials and Methods

### 2.1. Animals and Husbandry

All of the animals were housed according to EU [2] and national law [28,29]. In compliance with European Directive 2010/63/EC Article 1 5. (f) [30], the present study did not imply any invasive procedure or treatment to the animals.

The study was carried out on the research farm “Ruthe” of the University of Veterinary Medicine, Hannover, Germany, where a total of 3690 Lohmann Brown plus (LB+, conventional layer hybrids) and Lohmann Dual (LD, dual-purpose breeds) laying hens with intact beaks were housed according to the same standard management procedures [31]. The hens were kept in a conventional aviary system (Natura Nova 270, Big Dutchman, Vechta, Germany) consisting of two compartments with 926 and 927 LB+ each, and two compartments with 919 and 918 LD each. All of the animals were provided with alfalfa bales suspended in hay nets as standard enrichment material (about 200 hens per bale). At first signs of behavioural problems a graduated emergency scheme following the recommendations of the working group for laying hens at the Lower Saxony Ministry for Nutrition, Agriculture and Consumer protection [32] was applied (Table S1). Production parameters were continuously recorded by the farm staff.

### 2.2. Scoring Methods

VSc was performed on a weekly basis throughout the entire laying period (20th–71st week of life) containing 51 study days, on which the plumage and integument condition of more than 20,000 hens were assessed. Each week, a sample of 200 hens per genetic strain [27] was scored in five previously defined locations per compartment (*n* = 20 hens per location). The locations were chosen specifically for this barn ([13], modified) and included sections of the littered scratching areas, the intermediate ceiling of the aviary, the slats in front of the nest boxes, and the perches, respectively. For visual assessment, the hens’ body was divided into five regions: head/neck, back, tail, wing, and breast/belly ([26], modified). All of the body regions were scored for plumage and integument damage using a five and four point scale, respectively (Table 1). Concerning plumage condition, feather loss was ranked according to its degree, with no difference being made between the types of the respective feathers (flight feathers, other contour feathers, semiplumes). Integument damage was defined as fresh or crusted lesions affecting the skin only or also deeper tissues. Scars were not considered. Any other visible abnormalities, for instance lesions at the hens’ vent or toes, were recorded without a fixed scoring scheme.

In addition, HSc was carried out on seven study days (*n* = 60 hens per genetic strain and day) using the same scoring scheme as for the visual method. The dates for HSc (21st, 30th, 40th, 47th, 56th, 65th, and 70th week of life) were chosen according to a pilot study [21], in which particularly risk-bearing periods for the occurrence of feather pecking and cannibalism were identified. Both VSc and HSc were performed by the same observer on all of the study days. In the weeks in which both VSc and HSc were carried out, the observer started with the visual assessment. Once the entire sample was scored visually, HSc was conducted visiting each compartment of the aviary in a randomly different order to that in the VSc method.

### 2.3. Data Presentation and Statistical Analyses

All of the statistical analyses were performed using the software SPSS Statistics (version 24, IBM, Armonk, NY, USA). Calculations were carried out separately for plumage and integument damage, as well as for the five body regions of the hens. Since plumage and integument scores were interpreted as ordinally scaled variables, the proportions of the hens assigned to the respective scores were calculated for descriptive statistics. Accordingly, a non-parametric approach (Spearmans’s rho) was chosen for the comparison of scores given in VSc and HSc. The scores that were collected via the highly accurate, direct HSc method were therefore regarded as the gold standard.

## 3. Results

### 3.1. Production Data and Health Status

In the 70th week of life, the LB+, and LD hens weighed 2013 g and 2021 g on average. At the end of the laying period (71st week of life), the cumulative mortality amounted to 6.91% in the LB+ flocks, and 2.34% in the LD flocks, respectively. Feed consumption per hen and day ranged between 97.54 g (LD) and 116.92 g (LB+). In the LB+ flocks, egg production was 84.83% (71st week of life), whereas it was 73.56% in the LD flocks.

During the entire laying period, the health condition of both flocks was good. Neither the LB+ nor the LD hens needed any veterinary treatment.

### 3.2. Plumage and Integument Condition in LB+ and LD Hens

**Figure 1:**
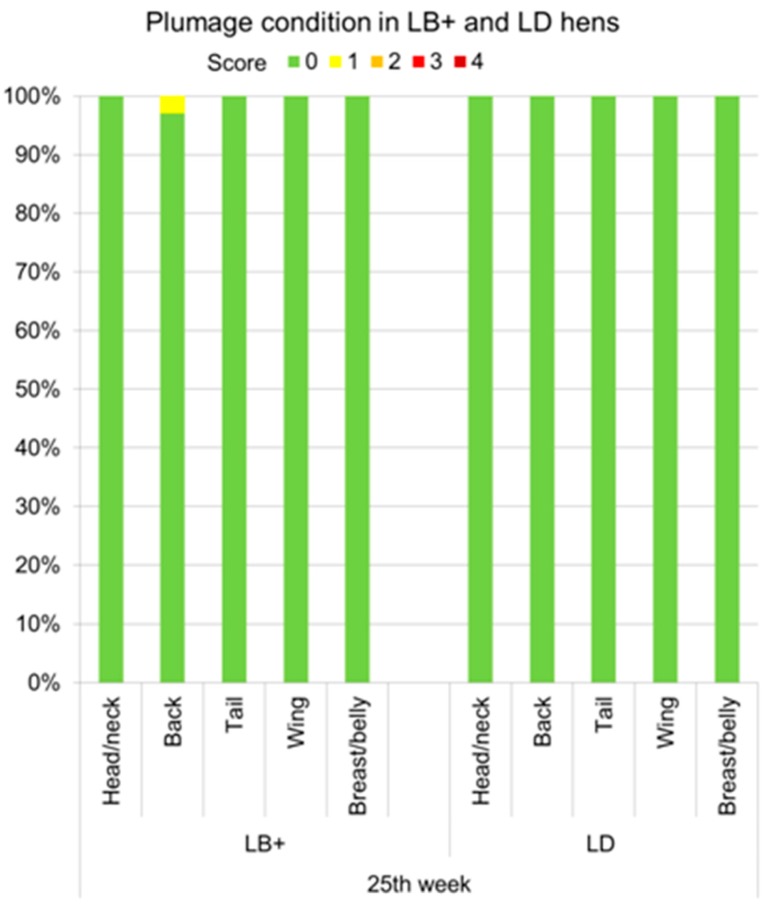
Proportions of Lohmann Brown plus (LB+) and Lohmann Dual (LD) hens (*n* = 400) with different plumage scores (0 (best) to 4 (worst)) for head/neck, back, tail, wing and breast/belly obtained by the Visual S**c**oring (VSc) method in the 25th week of life.

**Figure 2:**
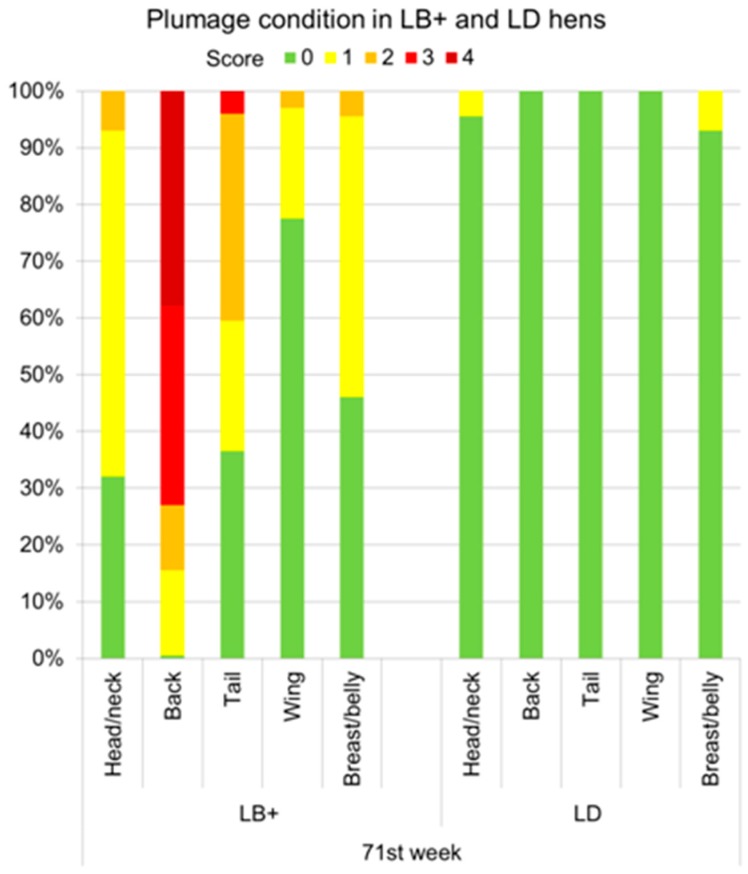
Proportions of LB+ and LD hens (*n* = 400) with different plumage scores (0 (best) to 4 (worst)) for head/neck, back, tail, wing, and breast/belly obtained by the VSc method in the 71st week of life.

Lesions indicating vent- or toe pecking were not observed by either of the two scoring methods during the whole study period. The entire proportions of LB+ and LD hens with different plumage and integument scores for five body regions obtained by the VSc and the HSc method are provided in the supplement (Tables S2–S9). Due to the large overall data set, in this section selected results are presented in detail to gain a closer insight into the development of plumage and integument damage in the flocks. Based on the more frequent observation intervals, these results relate to data obtained by the VSc method. In the 25th week of life, for instance, minor feather loss on the back (score 1) was found for the first time in 3% of the LB+ hens (Figure 1). At the same age, the plumage of all scored body regions of the LD was still intact. Plumage damage in the LB+ increased to the 71st week, in which all of the body parts were affected by feather loss to different extents (Figure 2). With 38% of the hens given the worst score of 4, the back was the most severely affected body region. Furthermore, 35%, 11.5%, and 15% of the hens showed lower degrees of feather loss (score 3, 2, and 1, respectively) on this body part. Only single hens (0.5% of the flock) were fully feathered on the back at the end of the laying period. The second most commonly affected body region of the LB+ hens was the tail. 0.5% of the animals showed almost denuded tails (score 4). The tails of 3.5%, 36.5%, and 23% of the LB+ hens were assigned to the plumage scores 3, 2, and 1, respectively. The plumage condition of the remaining body parts (head/neck, wing, and breast/belly) did not deteriorate to the extent of that of the back and the tail. These body regions received a maximum score of 2. On the head/neck and the breast/belly region minor feather loss (score 1) was observed in the majority of the LB+ hens (61% and 49.5%, respectively), whereas the feather covering of the wings was intact in most hens (77.5%). In contrast, only 4.5% and 7% of the LD hens showed minor feather loss on the head/neck and breast/belly region (score 1), which had started at the age of 40 weeks and remained almost constant until the end of the laying period. More severe plumage damage and feather loss on the back, tail, and wing were not found in the LD flocks.

**Figure 3:**
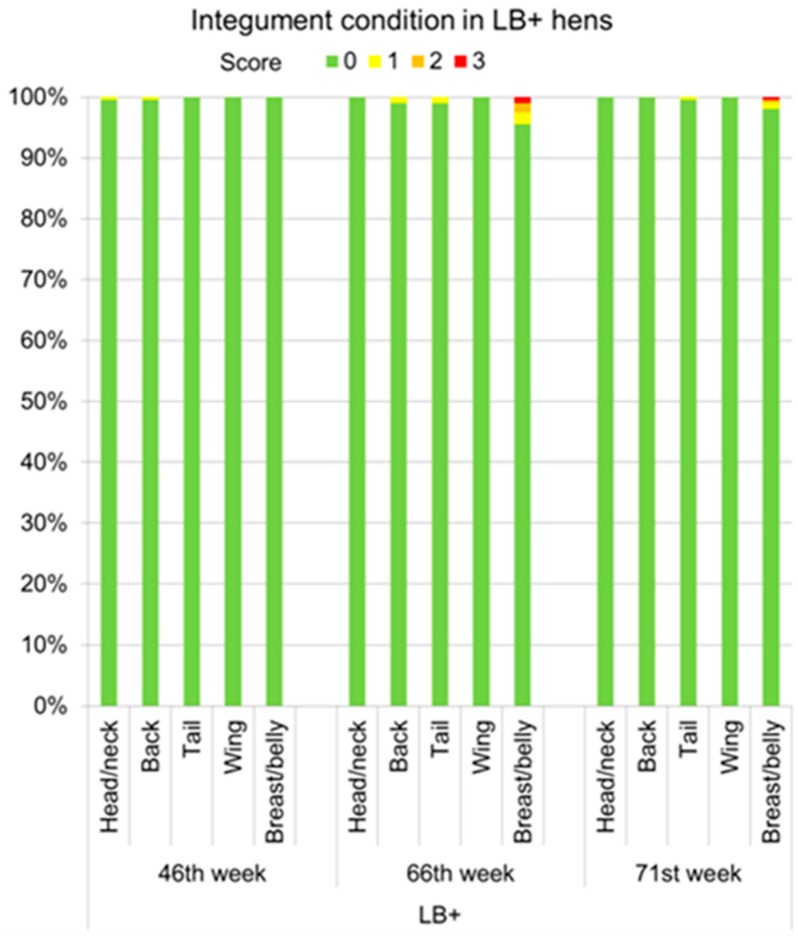
Proportions of LB+ hens (*n* = 200) with different integument scores (0 (best) to 4 (worst)) for head/neck, back, tail, wing, and breast/belly obtained by the VSc method in the 46th, 66th, and 71st week of life.

Integument damage in the form of single injuries <0.5 cm diameter or length (score 1) occurred in 0.5% of the LB+ hens on the head/neck and the back in the 46th week of life for the first time (Figure 3). The incidence of lesions reached a peak in week 66 when a total of 6% of the LB+ hens showed injuries of various degrees. At this age, the breast/belly was the most affected body part, on which severe integument damage (score 3) was found in 1% of the animals. Moreover, 1.5% and 2% of the LB+ had injuries corresponding to the scores 2 and 3, respectively, on the breast/belly region. Integument damage decreased to a total of 2.5% of injured LB+ hens at the end of the study (71st week of life). No injuries were detected in the LD hens by the VSc method throughout the laying period.

### 3.3. Plumage and Integument Score Comparisons between VSc and HSc Methods

**Figure 4:**
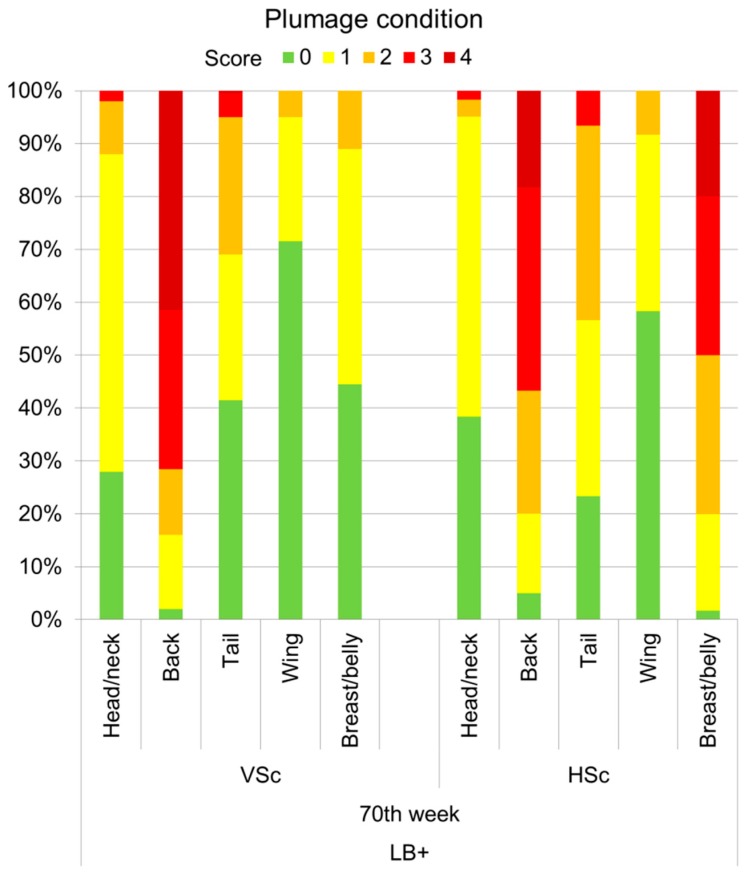
Proportions of LB+ hens with different plumage scores (0 (best) to 4 (worst)) for individual body regions given via VSc (*n* = 200 hens) and HSc (*n* = 60 hens) scoring methods in the 70th week of life.

For a concise overview, the scores that were obtained by VSc and HSc methods were compared descriptively for both hybrids for the end of the laying period when feather loss was most severe. Figure 4 shows the proportions of LB+ hens with different plumage scores for individual body regions obtained by the two scoring methods (70th week of life). The proportions of animals, in which feather loss in general (score 1–4) was observed, were similar in VSc and HSc for the body regions head/neck, back, and tail. According to results of VSc and HSc, the back was both the most commonly and most severely affected body part. However, with 41.5% more than twice as many, LB+ hens received the worst score of 4 in VSc as in HSc (18.4%) for this body region. In HSc, the backs of 38.3% and 23.3% of the hens were assigned to the plumage scores 3 and 2, respectively, whereas only 30% and 12.5% of the hens assessed in VSc were given these scores. With 14% and 15% similar proportions of LB+ hens with minor feather loss on the back (score 1) were detected by VSc and HSc. The breast/belly region of the LB+ hens, which was assigned to a maximum score of 2 during VSc, was scored worse in HSc. According to HSc scores, 20% of the hens received a score of 4, and only 1.7% of the LB+ showed intact plumage on the breast/belly region.

**Figure 5:**
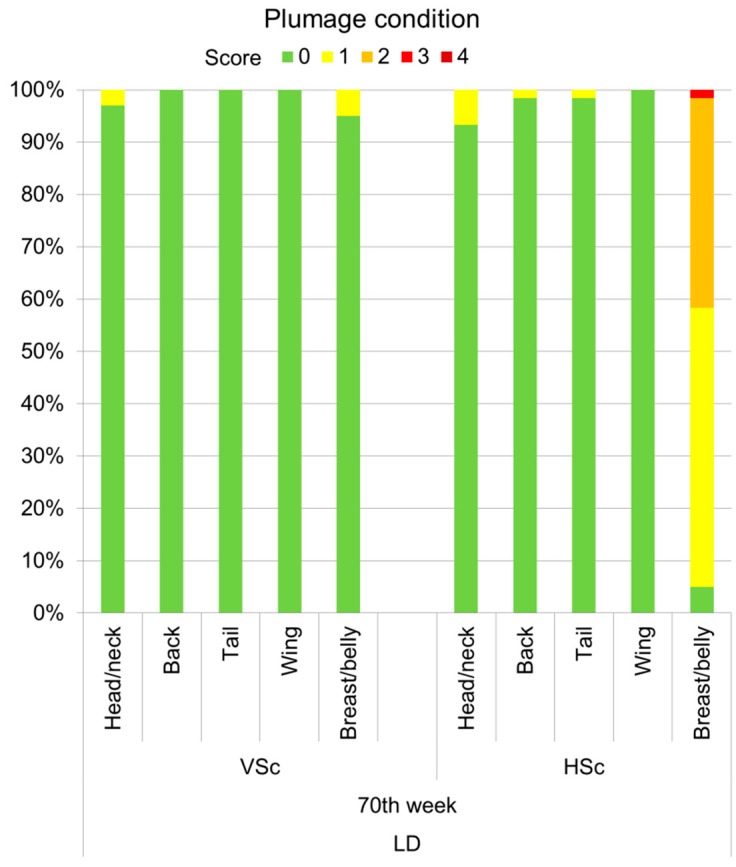
Proportions of LD hens with different plumage scores (0 (best) to 4 (worst)) for individual body regions given via VSc (*n* = 200 hens) and HSc (*n* = 60 hens) scoring methods in the 70th week of life.

Similar patterns applied to the descriptive comparison of plumage scores between VSc and HSc methods in the LD hens (Figure 5). In the majority of the LD (93.3–100%), no feather loss was detected by both the VSc and the HSc method, except for the breast/belly region. During VSc, only 5% of the animals were scored for minor plumage loss (score 1) on this body part, whereas score 1 was assigned to the majority of the hens (53.3%) in HSc. Furthermore, in HSc, 40% and 1.7% of the LD hens received the scores 2 and 3, respectively, for the breast/belly region. Only 5% of the LD hens showed intact plumage on the breast/belly in the close inspections (HSc) in the 70th week of life.

As expected from the descriptive comparisons, significant positive correlations between scores obtained by using VSc and HSc methods were found for most of the examined body regions and weeks in the LB+ hens (Table 2). The only exception was the breast/belly region. The scores for this body part did not correlate in most weeks, which is in line with the descriptive comparison at the end of the laying period (Figure 4). Perfect correlations (*r*_s_ = 1.00) between VSc and HSc scores occurred when the hens’ plumage was still intact, for instance, in the 21st week of life. In the LD hens, this applied to the majority of the tested weeks and body parts (Table 3), since feather loss was generally scarce in these hybrids. However, as in the LB+, the scores for the breast/belly region did not correlate between VSc and HSc methods from the 40th week of life. 

Since the incidence of injuries was generally low, calculations for the comparison of integument scores were carried out for the entire laying period (21st–70th week of life). The results that are presented in Table 4 show that there were strong positive correlations between VSc and HSc scores (*r_s_* = 0.86–1.00, *p* < 0.01), even for the breast/belly region in both of the hybrids (LB+: *r*_s_ = 0.86; LD: *r*_s_ = 0.97). In the LD hens, the correlation coefficients calculated for the head/neck (*r*_s_ = 0.97) and the breast/belly region (*r*_s_ = 0.97) indicate that although skin damage was not observed during VSc, sporadic injuries were detected by the HSc method (Table S9).

## 4. Discussion

In the present study, the plumage and integument condition of conventional layer hybrids and dual-purpose breeds was comparatively assessed at farm level by means of a mere visual method, and a scoring method, including the handling of individual animals.

Since plumage loss is a valid animal-based indicator for feather pecking in laying hens [26], the poor plumage condition of the LB+ flocks at the end of the laying period can be explained by this behavioural disorder. The most commonly and severely affected body regions were the back and the tail, which were almost denuded (> 75% of the feathers missing) in 38% and 0.5%, respectively, of the LB+ hens in the 71st week of life. This is in line with former investigations in which—due to a slightly different division of the hens’ body parts—the areas that were primarily prone to feather damages were the back and the rump [26], the rump and the tail [27], and the back, rump, and tail [33]. In addition, the breast/belly region was affected by feather loss in the majority of the LB+ hens (98.3%) when assessed by means of the close inspection method (HSc). This was also found by Bilcik and Keeling [26]. However, the extent of plumage damage on the breast/belly could not be related to the proportion of pecks that were directed at this body region [26]. The hens in the present study did not suffer from clinical diseases, such as diarrhea or other cloacal discharge, which can lead to plumage deterioration on the breast/belly region. Therefore, mechanical abrasion from the perches or slats of the aviary system might be a more likely explanation for the observed feather loss on this body part. In the LB+ hens, feather loss started at 25 weeks, and deteriorated with age. This time course of plumage damage is similar to that observed in other studies [1,34]. When compared to plumage damage, and thus to feather pecking, integument damage occurred with a delay of several weeks. Similar observations were made by Spindler et al. [21], and by Freytag et al. [35] supporting the hypothesis of severe feather pecking turning into tissue pecking once bald patches appear on the hens’ bodies [5]. Since plumage condition deteriorated continuously in the LB+ hens, the measures taken according to the emergency scheme [32], for instance, the provision of additional environmental enrichment materials (Table S1), did not appear to be fully sufficient to eradicate feather pecking. However, it was possible to calm down the situation concerning tissue pecking, which is in line with former investigations [21].

The slight feather loss on the head/neck in 4.5% of the LD hens may be rather due to aggressive pecks than to feather pecking behaviour [26]. Similar to the LB+ hens, feather loss on the breast/belly region in the LD hens may be attributable to mechanical abrasion. It is unlikely that the skin injuries, which were observed only sporadically in single LD hens during HSc, were related to extensive tissue pecking in these flocks. Since no direct behavioural observations were carried out in the present study, we cannot exclude the occurrence of gentle feather pecking in the LD hens, which usually does not result in feather loss [26]. However, severe behavioural disorders only occurred in the LB+ hens, though both genetic strains were kept under the same housing and management conditions in one building, including, for instance, the same feed, light quality and intensity, and staff. Therefore, genetic differences between the hybrids were likely responsible for the development and expression of damaging pecking behaviour [9]. Since LB+ hens were brown layers, and LD hens had white feathering, differences in plumage colouration may have accounted for the variation in plumage damage to a certain degree. Bright et al. [36] found significantly less plumage damage in white feathered hens than in black and grey birds of the same strain. Similar to our results, brown feathered laying hens (ISA brown) showed most plumage damage on the back, whereas in white feathered flocks (Dekalb White), mainly the neck and the belly were affected by feather loss [20]. However, in the present study, plumage colour effects were also confounded by layer strain. It was therefore not possible to separate these effects from other genetically determined strain differences, such as fearfulness or temperament. It should be further investigated to which extent the lower production performance of the LD hens affected the occurrence of behavioural problems. The results of former studies comparing commercial layer hybrids with lower yielding purebred lines are inconsistent. In one experiment, purebred New Hampshire hens showed significantly less feather pecking than commercial layer hybrids [25], whereas in another investigation, hens of the Danish landrace, which were manly bred for exhibition purposes, were more prone to damaging behaviour than high yielding hybrids [22]. Accordingly, the results by Hocking et al. [24] indicated that the expression of feather pecking and cannibalistic behaviour was highly breed specific but occurred in both traditional and commercial layer lines.

The visual method (VSc) for assessing the hens’ plumage and integument condition that is presented in this study combines several advantages of methods developed earlier [13,26,27]. Feather and plumage damage were scored separately for five body parts in order to mirror the different etiology of feather loss on these body regions [26]. In addition, whole body scores were not calculated, since—at least when whole body scores were presented as unweighted means [1]—the severity of feather loss on the back and tail of the LB+ hens would have been underestimated due to the relatively good feather condition of the wing throughout the laying period. Under field conditions, where it is not possible to score each animal individually, the chosen sample should represent the entire flock, which can be achieved by assessing birds in different functional areas of the stable [13]. This is particularly important for VSc, since without fixed scheme for choosing the animals to be assessed, observers would tend to see and score bald or injured animals first, and the results at the flock level would therefore be biased.

Similar to Bright et al. [27] we compared plumage and integument scores that were obtained by a VSc and an HSc method. In contrast to the experiment cited, in which the same focal hens were scored by means of the VSc and the HSc method, the present assessments were carried out at the flock level. This means that the animals scored by either of the two methods belonged to the same flock, but were not necessarily the same individuals. Nevertheless, strong positive correlations between the scores given in VSc and HSc were observed for the body regions that were mostly affected by feather pecking (back, tail) in both hybrids, and in the majority of the tested weeks (*r*_s_ > 0.90, *p* < 0.01). Perfect correlations (*r*_s_ = 1.00, *p* < 0.01) between scores occurred when the plumage and the integument of the hens were intact, and must therefore be interpreted with caution. However, these perfect correlations also indicate that there were no slight alterations of the plumage and the integument, which were missed in VSc and became only apparent during HSc. The present findings are in accordance with the investigations by Bright et al. [27], who found correlation coefficients for the plumage condition of all scored body parts ranging from 0.79 to 0.96. However, the breast/belly region, which was scored worse in HSc than in VSc in the present study, was not included. Since the breast/belly region was the only body part for which VSc and HSc scores did not correlate, this may be more likely caused by an underestimation in VSc than by the variations of the sample at the flock level. Although the animals were assessed in different locations of the barn, the breast/belly region was often not fully visible for the observer, for instance when the hens were perching. In addition, the thighs of the hens cover parts of the breast/belly. Thus, inaccuracies in VSc may be explained by the challenge to assess this body part visually. Conversely, integument damages on the breast/belly were identified reliably by the VSc method (*r*_s_ = 0.86–0.97, *p* < 0.01). This may be due to the fact that injuries mainly occurred once a body part was bald, and were then easily detectable via VSc. Additionally, the precise location of most of the injuries within the breast/belly region was next to the edges of the back and the tail region [21], and was therefore not covered by the hens’ thighs. Therefore, the definition of the breast/belly region should be reconsidered before further applying the presented VSc scheme. For future use of the VSc method, it should also be considered which purpose the assessed indicators should serve. Due to the different etiology of feather loss [26], not all body parts seem to be equally important to assess for certain purposes. To monitor feather pecking behaviour only, it might be sufficient to score the back and the tail of the hens. However, if the occurrence of for instance cloacal cannibalism should be detected, the vent region must be consequently added to the assessment protocol. The same principle applies if health issues, such as diarrhea, or damage caused by the housing system should be recorded.

Regular flock monitoring and surveillance by determining and evaluating animal-based indicators on-farm is not only recommended [4,27], but also required by law in Germany [28]. Farmers housing livestock must keep records of these assessments in order to document the compliance with the legal requirements (e.g., species appropriate housing of the animals) [28]. Therefore, reliable scoring methods, which are feasible under commercial conditions, can assist farmers in fulfilling the legal requirements, and thus in improving the welfare of their animals. Since scoring the animals by the same person will not always be possible in this context, the inter-observer reliability should be tested for the presented VSc method [27].

The mere visual method that is presented in this study offers a sound alternative for determining the plumage and integument condition in laying hens at flock level, both when the animals were highly—as the conventional layer hybrids—and slightly—as the dual-purpose breeds—affected by feather loss and integument damage. Developing a reliable scoring method that is scarcely disruptive for the animals, as well as feasible for regular flock monitoring or longitudinal on-farm research, has broad implications for laying hen welfare. Therefore, existing methods should be continuously reviewed and refined, and their advantages should be combined in order to comply with the current state of knowledge.

## 5. Conclusions

The plumage and integument condition of conventional layer hybrids (LB+) and dual-purpose breeds (LD) was assessed at farm level by means of a mere visual method, and a scientifically established hands-on scoring method. During the laying period, plumage damage increased in the LB+ hens, particularly on the back and the tail. In contrast, the LD hens showed only minor feather loss which remained constant throughout the laying period. In the LB+ hens, integument damage reached a peak in week 66, whereas injuries were found only sporadically in the LD hens. The visual scoring method was a valid non-intrusive alternative for detecting both slight and severe plumage and integument damage in the tested hybrids.

## Figures and Tables

**Table 1:** Description of the scoring scheme used for the assessment of plumage and integument condition in conventional layer hybrids and dual-purpose breeds.

Parameter/Score	Feather Loss	Integument Damage
0	No feather loss	No integument damage
1	≤25% of the feathers of the body part missing	Single injury of <0.5 cm diameter or length
2	>25% and ≤50% of the feathers of the body part missing	Multiple injuries of <0.5 cm or single injuries of >0.5 cm and ≤1.0 cm
3	>50% and ≤75% of the feathers of the body part missing	Single or multiple injuries of >1.0 cm
4	>75% of the feathers of the body part missing	-

**Table 2:** Spearman’s rho and the level of significance for plumage score comparisons between VSc and HSc methods for individual body regions in LB+ laying hens (21st–70th week of life).

Correlation VSc and HSc	Week of Life	Head/Neck	Back	Tail	Wing	Breast/Belly
LB+	21	1.00 **	1.00 **	1.00 **	1.00 **	1.00 **
	30	1.00 **	1.00 **	0.92 **	0.78 **	0.78 **
	40	0.93 **	0.77 **	0.83 **	1.00 **	0.04
	47	0.93 **	0.96 **	0.61	1.00 **	0.40
	56	0.91 **	0.70 *	0.99 **	0.97 **	0.26
	65	0.90 **	0.97 **	0.91 **	0.92 **	0.16
	70	0.97 **	0.55	0.64 *	0.99 **	0.52

* (*p* < 0.05), ** (*p* < 0.01).

**Table 3:** Spearman’s rho and the level of significance for plumage score comparisons between VSc and HSc methods for individual body regions in LD laying hens (21st–70th week of life).

Correlation VSc and HSc	Week of Life	Head/Neck	Back	Tail	Wing	Breast/Belly
LD	21	1.00 **	1.00 **	1.00 **	1.00 **	1.00 **
	30	1.00 **	1.00 **	1.00 **	1.00 **	0.78 **
	40	0.88 **	0.86 **	1.00 **	0.86 **	0.26
	47	0.78 **	1.00 **	1.00 **	1.00 **	0.18
	56	0.75 *	0.86 **	0.86 **	0.86 **	0.18
	65	0.93 **	1.00 **	1.00 **	1.00 **	0.40
	70	0.99 **	0.86 **	0.86 **	1.00 **	0.42

* (*p* < 0.05), ** (*p* < 0.01).

**Table 4:** Spearman’s rho and the level of significance for integument score comparisons between VSc and HSc methods for individual body regions in LB+ and LD laying hens throughout the laying period (21st–70th week of life).

Correlation VSc and HSc	Head/Neck	Back	Tail	Wing	Breast/Belly
LB+	0.97 **	0.87 **	0.89 **	0.97 **	0.86 **
LD	0.97 **	1.00 **	1.00 **	1.00 **	0.97 **

** (*p* < 0.01).

## Supplementary Materials

The following are available online at www.mdpi.com/2076-2615/7/12/97/s1. Table S1: Graduated emergency scheme following the recommendations of the working group for laying hens at the Lower Saxony Ministry for Nutrition, Agriculture and Consumer protection, Table S2: Proportions of LB+ hens (*n* = 200) with different plumage scores (0 (best) to 4 (worst)) for five body regions obtained by the VSc method, Table S3: Proportions of LB+ hens (*n* = 200) with different integument scores (0 (best) to 3 (worst)) for five body regions obtained by the VSc method, Table S4: Proportions of LB+ hens (*n* = 60) with different plumage scores (0 (best) to 4 (worst)) for five body regions obtained by the HSc method, Table S5: Proportions of LB+ hens (*n* = 60) with different integument scores (0 (best) to 3 (worst)) for five body regions obtained by the HSc method, Table S6: Proportions of LD hens (*n* = 200) with different plumage scores (0 (best) to 4 (worst)) for five body regions obtained by the VSc method, Table S7: Proportions of LD hens (*n* = 200) with different integument scores (0 (best) to 3 (worst)) for five body regions obtained by the VSc method, Table S8: Proportions of LD hens (*n* = 60) with different plumage scores (0 (best) to 4 (worst)) for five body regions obtained by the HSc method, Table S9: Proportions of LD hens (*n* = 60) with different integument scores (0 (best) to 3 (worst)) for five body regions obtained by the HSc method.
