# Supplementary material for: Assessment of Plumage and Integument Condition in Dual-Purpose Breeds and Conventional Layers
Source: Animals (Basel). 2017 Dec 12;7(12):97. doi: 10.3390/ani7120097 (PMC5742791; doi:10.3390/ani7120097)
Supplement: Supplementary file 1 [file animals-07-00097-s001.pdf]

animals

Supplementary materials

Assessment of Plumage and Integument Condition in Dual-Purpose Breeds and Conventional Layers

Mona Franziska Giersberg *, Birgit Spindler and Nicole Kemper

Table S1. Graduated emergency scheme following the recommendations of the working group for laying hens at the Lower Saxony Ministry for Nutrition, Agriculture and Consumer protection.

Behavioural problem	Level*	Measure	Details	Period	Application in the present study
Agitation in the flock	1	Applying table salt via drinking water	1 g NaCl/ l drinking water	3 days	-
	2	Applying magnesium via drinking water (e.g. Emgevet®)	1-2 ml Emgevet®/ l drinking water	3-5 days	60th week of life for 3 consecutive days
Feather pecking/ cannibalism	1	Supplying chopped straw in the litter	1-2 times/ day	Tapered when situation calmed down	29th – 40th week of life
	2	Supplying grain in the litter	5 g/ hen and day	Continuously until depopulation	40th – 71st week of life
	3	Providing succulent feed (apples/carrots)	2 baskets/ 1000 hens filled daily	Tapered when situation calmed down	47th week of life, for about 4 weeks
	4	Providing pecking blocks	1 pecking block/ 250 hens	Continuously until depopulation	48th – 71st week of life
	5	Light reduction	Last measure! 10% of original light intensity	Reduced light intensity is maintained until depopulation	Ø light intensity 20st week of life: 15 lux<br>1. reduction: 60th week, Ø 10 lux<br>2. reduction: 62nd week, Ø 7 lux<br>3. reduction: 65th week, Ø 6 lux until depopulation
Exclusive occurrence of cannibalism/ cloacal cannibalism	1	Applying essential amino acids via drinking water (e.g. Koniamin®)	0.5-1.0 ml/ l drinking water	3 days	-

*If the previous measure does not show sufficient effect, the next level should be applied.

Table S2. Proportions of LB+ hens (n= 200) with different plumage scores (0 (best) to 4 (worst)) for five body regions obtained by the VSc method.

Week of life	Plumage score	Body region				
		Head/neck	Back	Tail	Wing	Breast/belly
**21**	0	100.0	100.0	100.0	100.0	100.0
	1	0.0	0.0	0.0	0.0	0.0
	2	0.0	0.0	0.0	0.0	0.0
	3	0.0	0.0	0.0	0.0	0.0
	4	0.0	0.0	0.0	0.0	0.0
**30**	0	98.5	69.5	95.5	98.5	100.0
	1	1.5	30.5	4.5	1.5	0.0
	2	0.0	0.0	0.0	0.0	0.0
	3	0.0	0.0	0.0	0.0	0.0
	4	0.0	0.0	0.0	0.0	0.0
**40**	0	76.0	63.5	64.5	100.0	98.0
	1	23.0	36.5	35.5	0.0	2.0
	2	1.0	0.0	0.0	0.0	0.0
	3	0.0	0.0	0.0	0.0	0.0
	4	0.0	0.0	0.0	0.0	0.0
**47**	0	48.0	13.0	87.0	99.0	93.0
	1	45.0	56.0	13.0	1.0	6.0
	2	7.0	28.0	0.0	0.0	1.0
	3	0.0	3.0	0.0	0.0	0.0
	4	0.0	0.0	0.0	0.0	0.0
**56**	0	44.5	2.5	94.0	98.5	79.5
	1	51.0	14.0	6.0	1.5	18.5
	2	4.5	45.0	0.0	0.0	2.0
	3	0.0	38.5	0.0	0.0	0.0
	4	0.0	0.0	0.0	0.0	0.0
**65**	0	33.0	1.0	57.0	88.5	690.5
	1	59.0	11.5	31.5	11.0	36.5
	2	8.0	16.0	10.0	0.5	3.0
	3	0.0	60.0	1.5	0.0	0.0
	4	0.0	11.5	0.0	0.0	0.0
**70**	0	32.0	0.5	36.5	77.5	46.0
	1	61.0	15.0	23.0	19.5	49.5
	2	7.0	11.5	36.5	3.0	4.5
	3	0.0	35.0	3.5	0.0	0.0
	4	0.0	38.0	0.5	0.0	0.0

Table S3. Proportions of LB+ hens (n= 200) with different integument scores (0 (best) to 3 (worst)) for five body regions obtained by the VSc method.

Week of life	Integument score	Body region				
		Head/neck	Back	Tail	Wing	Breast/belly
21	0	100.0	100.0	100.0	100.0	100.0
	1	0.0	0.0	0.0	0.0	0.0
	2	0.0	0.0	0.0	0.0	0.0
	3	0.0	0.0	0.0	0.0	0.0
30	0	100.0	100.0	100.0	100.0	100.0
	1	0.0	0.0	0.0	0.0	0.0
	2	0.0	0.0	0.0	0.0	0.0
	3	0.0	0.0	0.0	0.0	0.0
40	0	100.0	100.0	100.0	100.0	100.0
	1	0.0	0.0	0.0	0.0	0.0
	2	0.0	0.0	0.0	0.0	0.0
	3	0.0	0.0	0.0	0.0	0.0
47	0	100.0	99.5	100.0	100.0	100.0
	1	0.0	0.5	0.0	0.0	0.0
	2	0.0	0.0	0.0	0.0	0.0
	3	0.0	0.0	0.0	0.0	0.0
56	0	100.0	99.5	100.0	100.0	100.0
	1	0.0	0.5	0.0	0.0	0.0
	2	0.0	0.0	0.0	0.0	0.0
	3	0.0	0.0	0.0	0.0	0.0
65	0	100.0	98.5	99.5	100.0	96.0
	1	0.0	1.5	0.5	0.0	0.5
	2	0.0	0.0	0.0	0.0	2.5
	3	0.0	0.0	0.0	0.0	1.0
70	0	100.0	100.0	98.5	100.0	98.5
	1	0.0	0.0	0.5	0.0	1.0
	2	0.0	0.0	0.0	0.0	0.5
	3	0.0	0.0	1.0	0.0	0.0

Table S4. Proportions of LB+ hens (n= 60) with different plumage scores (0 (best) to 4 (worst)) for five body regions obtained by the HSc method.

Week of life	Plumage score	Body region				
		Head/neck	Back	Tail	Wing	Breast/belly
**21**	0	100.0	100.0	100.0	100.0	100.0
	1	0.0	0.0	0.0	0.0	0.0
	2	0.0	0.0	0.0	0.0	0.0
	3	0.0	0.0	0.0	0.0	0.0
	4	0.0	0.0	0.0	0.0	0.0
**30**	0	93.3	70.0	98.3	100.0	40.0
	1	6.7	30.0	1.7	0.0	30.0
	2	0.0	0.0	0.0	0.0	0.0
	3	0.0	0.0	0.0	0.0	0.0
	4	0.0	0.0	0.0	0.0	0.0
**40**	0	63.3	36.7	95.0	100.0	8.3
	1	35.0	60.0	5.0	0.0	46.7
	2	1.7	3.3	0.0	0.0	33.3
	3	0.0	0.0	0.0	0.0	11.7
	4	0.0	0.0	0.0	0.0	0.0
**47**	0	60.0	16.7	98.3	96.7	3.3
	1	33.3	51.7	0.0	3.3	63.3
	2	6.7	26.6	1.7	0.0	30.0
	3	0.0	5.0	0.0	0.0	3.35
	4	0.0	0.0	0.0	0.0	0.0
**56**	0	46.7	3.3	85.0	93.3	6.7
	1	51.7	33.3	15.0	6.7	30.0
	2	1.6	38.4	0.0	0.0	50.0
	3	0.0	21.7	0.0	0.0	13.3
	4	0.0	3.3	0.0	0.0	0.0
**65**	0	36.7	3.3	40.0	70.0	3.3
	1	50.0	16.7	41.7	26.7	26.7
	2	8.3	25.0	13.3	3.3	31.7
	3	5.0	45.0	5.0	0.0	33.3
	4	0.0	10.0	0.0	0.0	5.0
**70**	0	38.3	5.0	23.3	58.3	1.7
	1	56.7	15.0	33.3	33.4	18.3
	2	3.3	23.3	36.7	8.3	30.0
	3	1.7	38.3	6.7	0.0	30.0
	4	0.0	18.4	0.0	0.0	20.0

Table S5. Proportions of LB+ hens (n= 60) with different integument scores (0 (best) to 3 (worst)) for five body regions obtained by the HSc method.

Week of life	Integument score	Body region				
		Head/neck	Back	Tail	Wing	Breast/belly
**21**	0	100.0	100.0	100.0	100.0	100.0
	1	0.0	0.0	0.0	0.0	0.0
	2	0.0	0.0	0.0	0.0	0.0
	3	0.0	0.0	0.0	0.0	0.0
**30**	0	100.0	100.0	100.0	100.0	100.0
	1	0.0	0.0	0.0	0.0	0.0
	2	0.0	0.0	0.0	0.0	0.0
	3	0.0	0.0	0.0	0.0	0.0
**40**	0	100.0	98.3	100.0	100.0	100.0
	1	0.0	1.7	0.0	0.0	0.0
	2	0.0	0.0	0.0	0.0	0.0
	3	0.0	0.0	0.0	0.0	0.0
**47**	0	100.0	75.0	100.0	100.0	100.0
	1	0.0	20.0	0.0	0.0	0.0
	2	0.0	5.0	0.0	0.0	0.0
	3	0.0	0.0	0.0	0.0	0.0
**56**	0	98.3	75.0	98.3	100.0	95.0
	1	1.7	21.7	1.7	0.0	3.3
	2	0.0	3.3	0.0	0.0	1.7
	3	0.0	0.0	0.0	0.0	0.0
**65**	0	100.0	71.7	86.7	98.3	68.3
	1	0.0	23.3	11.6	1.7	25.0
	2	0.0	3.3	1.7	0.0	1.7
	3	0.0	1.7	0.0	0.0	0.0
**70**	0	100.0	858.0	96.7	100.0	73.3
	1	0.0	11.6	3.3	0.0	16.7
	2	0.0	1.7	0.0	0.0	5.0
	3	0.0	1.7	0.0	0.0	5.0

Table S6. Proportions of LD hens (n= 200) with different plumage scores (0 (best) to 4 (worst)) for five body regions obtained by the VSc method.

Week of life	Plumage score	Body region				
		Head/neck	Back	Tail	Wing	Breast/belly
21	0	100.0	100.0	100.0	100.0	100.0
	1	0.0	0.0	0.0	0.0	0.0
	2	0.0	0.0	0.0	0.0	0.0
	3	0.0	0.0	0.0	0.0	0.0
	4	0.0	0.0	0.0	0.0	0.0
30	0	100.0	100.0	100.0	100.0	100.0
	1	0.0	0.0	0.0	0.0	0.0
	2	0.0	0.0	0.0	0.0	0.0
	3	0.0	0.0	0.0	0.0	0.0
	4	0.0	0.0	0.0	0.0	0.0
40	0	98.0	100.0	100.0	100.0	100.0
	1	2.0	0.0	0.0	0.0	0.0
	2	0.0	0.0	0.0	0.0	0.0
	3	0.0	0.0	0.0	0.0	0.0
	4	0.0	0.0	0.0	0.0	0.0
47	0	95.5	100.0	100.0	100.0	100.0
	1	4.5	0.0	0.0	0.0	0.0
	2	0.0	0.0	0.0	0.0	0.0
	3	0.0	0.0	0.0	0.0	0.0
	4	0.0	0.0	0.0	0.0	0.0
56	0	96.5	100.0	100.0	100.0	100.0
	1	2.5	0.0	0.0	0.0	0.0
	2	1.0	0.0	0.0	0.0	0.0
	3	0.0	0.0	0.0	0.0	0.0
	4	0.0	0.0	0.0	0.0	0.0
65	0	92.5	100.0	100.0	99.5	97.5
	1	6.0	0.0	0.0	0.5	2.5
	2	1.5	0.0	0.0	0.0	0.0
	3	0.0	0.0	0.0	0.0	0.0
	4	0.0	0.0	0.0	0.0	0.0
70	0	97.0	100.0	100.0	100.0	95.0
	1	3.0	0.0	0.0	0.0	5.0
	2	0.0	0.0	0.0	0.0	0.0
	3	0.0	0.0	0.0	0.0	0.0
	4	0.0	0.0	0.0	0.0	0.0

Table S7. Proportions of LD hens (n= 200) with different integument scores (0 (best) to 3 (worst)) for five body regions obtained by the VSc method.

Week of life	Integument score	Body region				
		**Head/neck**	**Back**	**Tail**	**Wing**	**Breast/belly**
**21**	0	100.0	100.0	100.0	100.0	100.0
	1	0.0	0.0	0.0	0.0	0.0
	2	0.0	0.0	0.0	0.0	0.0
	3	0.0	0.0	0.0	0.0	0.0
**30**	0	100.0	100.0	100.0	100.0	100.0
	1	0.0	0.0	0.0	0.0	0.0
	2	0.0	0.0	0.0	0.0	0.0
	3	0.0	0.0	0.0	0.0	0.0
**40**	0	100.0	100.0	100.0	100.0	100.0
	1	0.0	0.0	0.0	0.0	0.0
	2	0.0	0.0	0.0	0.0	0.0
	3	0.0	0.0	0.0	0.0	0.0
**47**	0	100.0	100.0	100.0	100.0	100.0
	1	0.0	0.0	0.0	0.0	0.0
	2	0.0	0.0	0.0	0.0	0.0
	3	0.0	0.0	0.0	0.0	0.0
**56**	0	100.0	100.0	100.0	100.0	100.0
	1	0.0	0.0	0.0	0.0	0.0
	2	0.0	0.0	0.0	0.0	0.0
	3	0.0	0.0	0.0	0.0	0.0
**65**	0	100.0	100.0	100.0	100.0	100.0
	1	0.0	0.0	0.0	0.0	0.0
	2	0.0	0.0	0.0	0.0	0.0
	3	0.0	0.0	0.0	0.0	0.0
**70**	0	100.0	100.0	100.0	100.0	100.0
	1	0.0	0.0	0.0	0.0	0.0
	2	0.0	0.0	0.0	0.0	0.0
	3	0.0	0.0	0.0	0.0	0.0

Table S8. Proportions of LD hens (n= 60) with different plumage scores (0 (best) to 4 (worst)) for five body regions obtained by the HSc method.

Week of life	Plumage score	Body region				
		Head/neck	Back	Tail	Wing	Breast/belly
**21**	0	100.0	100.0	100.0	100.0	100.0
	1	0.0	0.0	0.0	0.0	0.0
	2	0.0	0.0	0.0	0.0	0.0
	3	0.0	0.0	0.0	0.0	0.0
	4	0.0	0.0	0.0	0.0	0.0
**30**	0	100.0	100.0	100.0	100.0	91.7
	1	0.0	0.0	0.0	0.0	8.3
	2	0.0	0.0	0.0	0.0	0.0
	3	0.0	0.0	0.0	0.0	0.0
	4	0.0	0.0	0.0	0.0	0.0
**40**	0	98.3	98.3	100.0	98.3	23.3
	1	1.7	1.7	0.0	1.7	45.0
	2	0.0	0.0	0.0	0.0	25.0
	3	0.0	0.0	0.0	0.0	1.7
	4	0.0	0.0	0.0	0.0	0.0
**47**	0	100.0	100.0	100.0	100.0	10.0
	1	0.0	0.0	0.0	0.0	45.0
	2	0.0	0.0	0.0	0.0	25.0
	3	0.0	0.0	0.0	0.0	1.7
	4	0.0	0.0	0.0	0.0	0.0
**56**	0	95.0	96.7	98.3	98.3	11.7
	1	5.0	3.3	1.7	1.7	75.0
	2	0.0	0.0	0.0	0.0	11.6
	3	0.0	0.0	0.0	0.0	1.7
	4	0.0	0.0	0.0	0.0	0.0
**65**	0	91.7	100.0	100.0	98.3	6.7
	1	5.0	0.0	0.0	1.7	50.0
	2	3.3	0.0	0.0	0.0	43.3
	3	0.0	0.0	0.0	0.0	0.0
	4	0.0	0.0	0.0	0.0	0.0
**70**	0	93.3	98.3	98.3	100.0	5.0
	1	6.7	1.7	1.7	0.0	53.3
	2	0.0	0.0	0.0	0.0	40.0
	3	0.0	0.0	0.0	0.0	1.7
	4	0.0	0.0	0.0	0.0	0.0

Table S9. Proportions of LD hens (n= 60) with different integument scores (0 (best) to 3 (worst)) for five body regions obtained by the HSc method.

Week of life	Integument score	Body region				
		Head/neck	Back	Tail	Wing	Breast/belly
**21**	0	100.0	100.0	100.0	100.0	100.0
	1	0.0	0.0	0.0	0.0	0.0
	2	0.0	0.0	0.0	0.0	0.0
	3	0.0	0.0	0.0	0.0	0.0
**30**	0	100.0	100.0	100.0	100.0	100.0
	1	0.0	0.0	0.0	0.0	0.0
	2	0.0	0.0	0.0	0.0	0.0
	3	0.0	0.0	0.0	0.0	0.0
**40**	0	100.0	100.0	100.0	100.0	100.0
	1	0.0	0.0	0.0	0.0	0.0
	2	0.0	0.0	0.0	0.0	0.0
	3	0.0	0.0	0.0	0.0	0.0
**47**	0	100.0	100.0	100.0	100.0	100.0
	1	0.0	0.0	0.0	0.0	0.0
	2	0.0	0.0	0.0	0.0	0.0
	3	0.0	0.0	0.0	0.0	0.0
**56**	0	100.0	100.0	100.0	100.0	100.0
	1	0.0	0.0	0.0	0.0	0.0
	2	0.0	0.0	0.0	0.0	0.0
	3	0.0	0.0	0.0	0.0	0.0
**65**	0	98.3	100.0	100.0	100.0	98.3
	1	1.7	0.0	0.0	0.0	1.7
	2	0.0	0.0	0.0	0.0	0.0
	3	0.0	0.0	0.0	0.0	0.0
**70**	0	100.0	100.0	100.0	100.0	100.0
	1	0.0	0.0	0.0	0.0	0.0
	2	0.0	0.0	0.0	0.0	0.0
	3	0.0	0.0	0.0	0.0	0.0
